# Fabrications and Performance of Wireless LC Pressure Sensors through LTCC Technology

**DOI:** 10.3390/s18020340

**Published:** 2018-01-25

**Authors:** Lin Lin, Mingsheng Ma, Faqiang Zhang, Feng Liu, Zhifu Liu, Yongxiang Li

**Affiliations:** 1 CAS Key Laboratory of Inorganic Functional Materials and Devices, Shanghai Institute of Ceramics, 1295 Dingxi Road, Shanghai 200050, China; linlin@student.sic.ac.cn (L.L.); zhangfq@mail.sic.ac.cn (F.Z.); liuf@mail.sic.ac.cn (F.L.); yxli@mail.sic.ac.cn (Y.L.); 2 University of Chinese Academy of Sciences, 19 Yuquan Road, Beijing 100049, China; 3 School of Engineering, RMIT University, Melbourne, VIC 3001, Australia

**Keywords:** wireless pressure sensor, LTCC, LC resonator, cavity

## Abstract

This paper presents a kind of passive wireless pressure sensor comprised of a planar spiral inductor and a cavity parallel plate capacitor fabricated through low-temperature co-fired ceramic (LTCC) technology. The LTCC material with a low Young’s modulus of ~65 GPa prepared by our laboratory was used to obtain high sensitivity. A three-step lamination process was applied to construct a high quality cavity structure without using any sacrificial materials. The effects of the thickness of the sensing membranes on the sensitivity and detection range of the pressure sensors were investigated. The sensor with a 148 μm sensing membrane showed the highest sensitivity of 3.76 kHz/kPa, and the sensor with a 432 μm sensing membrane presented a high detection limit of 2660 kPa. The tunable sensitivity and detection limit of the wireless pressure sensors can meet the requirements of different scenes.

## 1. Introduction

The detection of pressure variations is in demand in a variety of fields, including the automotive industry, robotics, and biomedicine. [1,2,3]. Passive wireless pressure sensors have attracted attention because they can bypass cable connections and the application of a power source [4]. Wireless LC pressure sensors are composed of a planar spiral inductor and a cavity capacitor whose capacitance can vary according to strain by stress (pressure) [5]. The resonating frequency of the LC sensor is pressure-dependent and can be detected remotely by measuring the impedance of an external coupled antenna as a function of frequency [6]. However, most wireless LC sensors are based on silicon and polymer substrate materials, which do not meet pressure measurement requirements in extreme environments with, for example, high temperature, high radiation, and chemical corrosion [7,8].

Low temperature co-fired ceramic (LTCC) technology is considered as a promising approach for wireless pressure sensor fabrication due to its feasibility for the construction of three-dimensional ceramic structures with embedded cavities and circuits. In particular, the good thermal, mechanical, and electrical properties of LTCC materials as well as its compatibility with thick film hybrid technology make it suitable for pressure sensors [9,10,11]. High-temperature wireless LC pressure sensors based on LTCC technology were firstly reported by Allen et al. [12]. After that, Radosavljevic and Xiong et al. improved the performance of the sensors by optimizing the sensor structures [13,14]. According to the working mechanism of the cavity capacitive pressure sensor, the sensitivity of the pressure sensor is inversely proportional to the Young’s modulus of the LTCC material [13]. A lower Young’s modulus may lead to higher sensitivity. Thus, the property of the LTCC material is one of the keys to obtaining high-performance wireless LC pressure sensors.

In this work, a kind of LTCC material with a low Young’s modulus of ~65 GPa, which is about only half of that of DuPont 951 (DuPont, Wilmington, DE, USA), was used to fabricate the wireless LC pressure sensors. A three-step lamination process was applied to construct the cavity structure without using any sacrificial materials. The effects of LTCC membrane thickness on the sensitivity of pressure sensors were investigated.

## 2. Sensor Design

**Figure 1:**
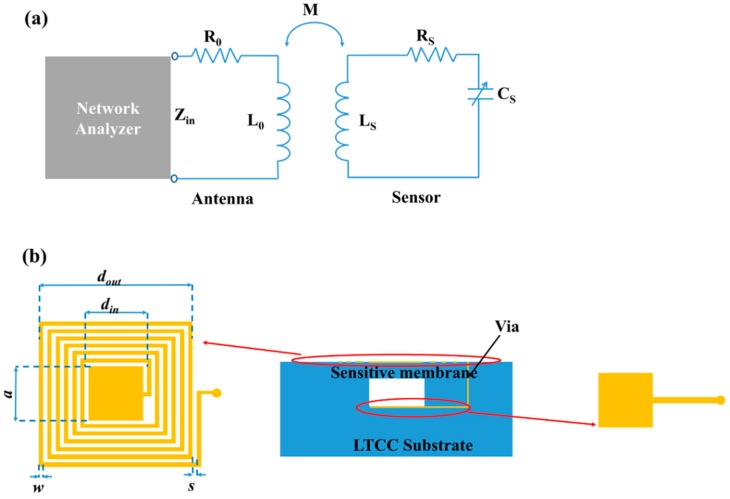
(**a**) Equivalent circuit of inductive coupling between the sensor and the reader antenna. (**b**) Schematic of the wireless LTCC pressure sensor.

The model of the wireless LC pressure sensing have been well established. As shown in Figure 1a, the wireless LTCC pressure sensor is equivalent to an LC circuit. *L_S_* and *C_S_* are a planar spiral inductor and a cavity capacitor, as shown in Figure 1b. The resonant frequency of the LC circuit *f_S_* can be represented as

(1) $$f_{S}=\frac{1}{2\pi \sqrt{L_{S}C_{S}}}.$$

For a square planar inductor, the *L_S_* value depends on the structural dimensions of the planar spiral inductor [15]:

(2) $$L_{S}=1.39\times 10^{-6}\left(d_{out}+d_{in}\right)\log\left(4\frac{d_{out}+d_{in}}{d_{out}-d_{in}}\right)$$

where *d_out_* and *d_in_* are the external and internal dimensions of the planar spiral inductor, respectively. The initial capacitance of parallel capacitor can be expressed as [12]

(3) $$C_{0}=\frac{\varepsilon_{0}ab}{t_{g}+\frac{t_{m}}{\varepsilon_{r}}}$$

where $\varepsilon_{0}$ is the free space permittivity (8.85 × 10^−12^ F/m), and $\varepsilon_{r}$ is the relative dielectric constant of the LTCC material (6.2) [16]. *t_g_* is the height of the embedded cavity, and *t_m_* is the thickness of pressure sensitive membrane. The parameters of the designed LC pressure sensor are summarized in Table 1.

Due to the variation of the *C_S_* value under an external pressure, the resonant frequency *f_S_* of the LC circuit is pressure-dependent and can be detected wirelessly by measuring the impedance variation of a reader antenna. The equivalent input impedance (*Z_in_*) of the reader antenna can be expressed as [17]

(4) $$Z_{in}=R_{0}+j2\pi L_{0}\left[1+\frac{k^{2}{\left(\frac{f}{f_{S}}\right)}^{2}}{1+\frac{j}{Q}\left(\frac{f}{f_{S}}\right)-{\left(\frac{f}{f_{S}}\right)}^{2}}\right]=F\left(f\right)$$

where *f_S_* and *Q* are the resonant frequency and the quality factor of the LC circuit, respectively. *k* is the coupling coefficient between the LC sensor and reader antenna. The reflection loss *S*_11_ of the coupling signal can be expressed as [17]:

(5) $$S_{11}=\frac{Z_{in}-Z_{0}}{Z_{in}+Z_{0}}{|}_{Z_{0}=50\;\Omega }$$

where *Z*_0_ is the intrinsic impedance of the signal emitter, with a fixed value of 50 Ω.

According to Equations (4) and (5), the external reader antenna can form strong inductive coupling with the LC sensor when *f* = *f_S_*, and the magnitude of *S*_11_ parameter drops to the minimum at the resonant frequency *f*_0_, so that the sensing signal can be wirelessly detected [18]. 

On the other hand, the deformation of sensitive membrane under a pressure can be evaluated according to the membrane deformation theory [19]. The membrane deformation model assumes that the maximum deformation *d*_0_ is smaller than its thickness *t_m_* (*d* ≤ 0.2*t_m_*). For a flat rectangular diaphragm, the center deflection of the membrane *d*_0_ with applied pressure *P* is described by Equation (6):

(6) $$d_{0}=\frac{0.00126Pa^{4}*12\left(1-v^{2}\right)}{E{\left(2t_{m}\right)}^{3}}$$

where *v* is the Poisson’s ratio, *t_m_* is the thickness of the diaphragm, and *E* is the Young’s modulus of the sensing membrane material. The force field simulation of sensitive membrane under pressure was conducted using ANSYS software.

**Figure 2:**
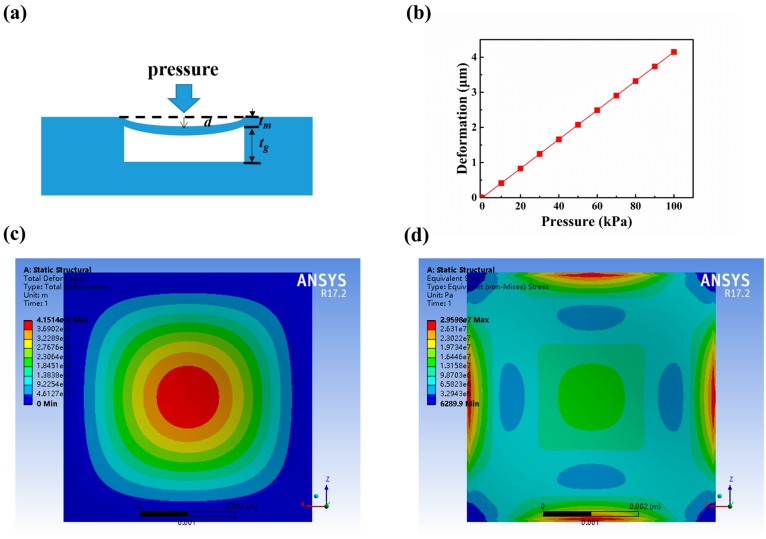
(**a**) Schematic diagram of the thin plate deformation model. (**b**) The sensing membrane deformation versus the applied pressure. (**c**) The simulated result of the sensing membrane deformation under 100 kPa pressure. (**d**) The simulated result of the equivalent stress distribution in sensing membrane under 100 kPa pressure.

In the simulation, the pressures in the range of 0–100 kPa were applied. The diaphragm thickness *t_m_* = 148 µm. The cavity length, width, and height were 6.5 mm, 6.5 mm, and 395 μm respectively. Figure 2b shows the deformation curve of the membrane as a function of the applied pressure. It can be seen that the deformation increase linearly with the increase in applied pressure. Figure 2c,d showed the deformation of the sensing membrane and the stress distribution in the sensing membrane under 100 kPa. The equivalent (von-mises) stress at the edge of the membrane is 29.598 MPa, which is much lower than the flexural strength of the LTCC substrate material (200 MPa). This means that the membrane will not be damaged under a wide range of applied pressure.

## 3. Pressure Sensor Fabrication and Test

The details about the LTCC green tapes SICCAS-K5F3 (SICCAS, Shanghai, China) fabrication can be found in our previous paper [16]. The parameters of SICCAS-K5F3 and DuPont 951 materials are listed in Table 2. The SICCAS-K5F3 LTCC material has a low Young’s modulus of 65 GPa, which is much lower than that of DuPont 951 (120 GPa). The low Young’s modulus would be good for obtaining high pressure sensitivity [13].

**Figure 3:**
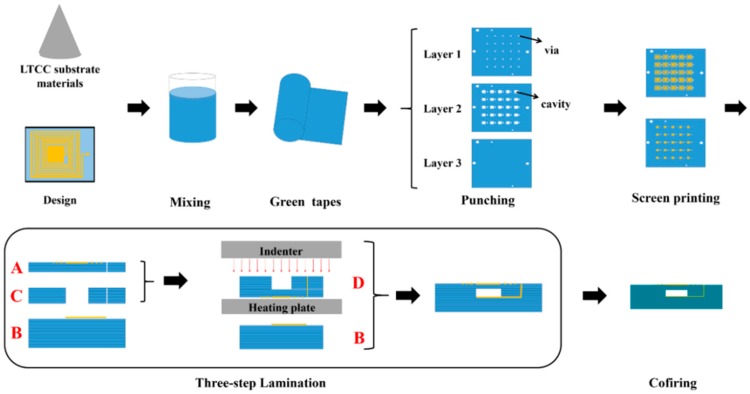
Fabrication process of the wireless LTCC pressure sensors.

Figure 3 shows the fabrication process of the wireless LTCC pressure sensor. The LTCC green tapes were cut and vias punched (PAM-4S, KEKO, Zuzemberk, Slovenia) according to the designed patterns. Silver paste (LL612, DuPont, Wilmington, DE, USA) was used for LC antenna preparation by screen-printing (P-200A, KEKO, Zuzemberk, Slovenia). The construction of uniform cavity is considered as the most important process for fabricating the pressure sensor. We used a modified three-step lamination process to fabricate cavity structure without any sacrificial inserts. As schematically shown in Figure 3, firstly, the LTCC green tapes with different layers were stacked to form the sensing membranes (Part A), the bottom substrate (Part B), and the cavity structure (Part C), respectively. The laminating pressure was 5 MPa, and the laminating temperature was 60 °C with a holding time of 120 s. Secondly, Parts A and C were stacked together to form Part D by using the same laminating conditions mentioned above. Finally, Parts B and D were laminated together at the optimized laminating temperature and time (55 °C, 60 s) to form the cavity. The wireless pressure sensors were obtained by heating the green samples initially at 450 °C for 120 min to remove the binders and then sintered at 900 °C for 30 min in air. The pressure sensors with different sensing membrane thicknesses of 3, 5, 7, and 9 layers were fabricated, and they are denoted as S3, S5, S7 and S9, respectively. All these sensors have the same thicknesses in Parts B and C. The cross-section of the cavity structure fabricated under different conditions was observed with a scanning electron microscope (SEM, ZEISS Supra55, Jena, Germany).

**Figure 4:**
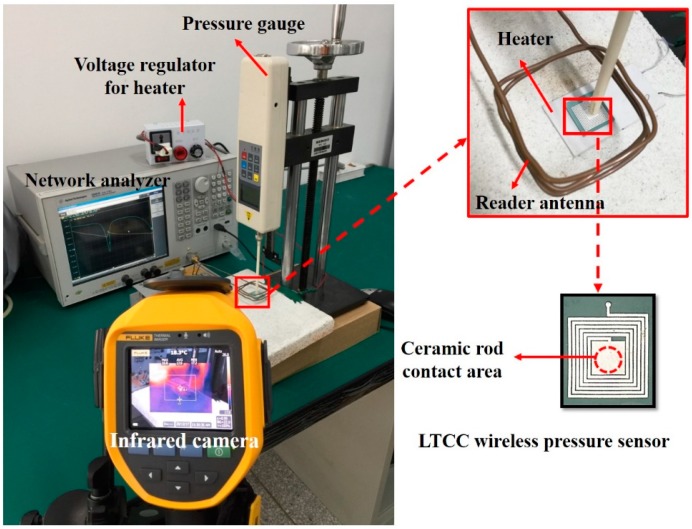
Photograph of the test system for temperature and pressure sensing measurement.

Figure 4 shows the photograph of the system for temperature and pressure sensing measurement. A homemade reader antenna was connected to a vector network analyzer (E5061B, Agilent, Santa Clara, CA, USA) and placed 10 mm above the sensor. The radio wave reflected from the LC pressure sensor was detected using this antenna. The RF reflection coefficient (S11) and corresponding *f*_0_ were recorded by the network analyzer. A digital force gauge (HLB, Ai-debao, Shenzhen, China) connected with an alumina rod (diameter = 4.25 mm) is placed on the upper electrode of the integrated cavity capacitor of the LTCC pressure sensor. The pressure value can be calculated by the relationship between the applied force and the contact area on the sensor. Wireless pressure sensors were also tested at elevated temperature. For high temperature measurement, the sensor was placed on the flat surface of a heater, and the heating temperature is controlled by a voltage regulator. The infrared thermal camera (Ti400, Fluke, Everett, WA, USA) was used to monitor the heating temperature.

## 4. Results and Discussion

**Figure 5:**
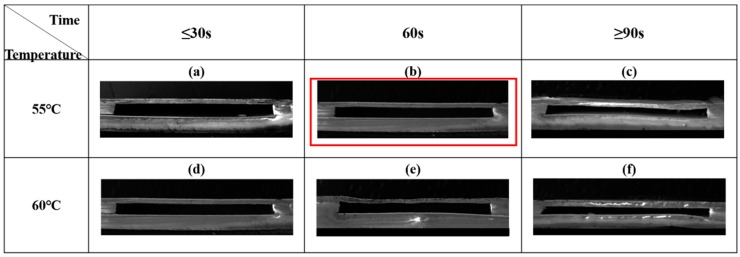
SEM images of the cross-section of cavities under different laminating conditions: (**a**) T=55 ℃, t≤30 s; (**b**) T=55 ℃, t=60 s; (**c**) T=55 ℃, t≥90 s; (**d**) T=60 ℃, t≤30 s; (**e**) T=60 ℃, t=60 s; (**f**) T=60 ℃, t≥90 s.

The microstructure of the pressure sensors was investigated to check the validity of the fabrication process. Figure 5 shows the SEM pictures of the cross-sections of the cavity structure fabricated under different conditions. It can be seen that the best laminating condition is 55 °C with a holding time of 60 s. As shown in Figure 5b, a uniform cavity structure could be obtained under this condition. For all samples, the same laminating conditions were used for the first and the second lamination steps, which could avoid the crack of sub-layers. It was considered that a higher laminating temperature leads to a better adhesion of LTCC green tapes [20,21,22]. Nevertheless, the high temperature could also damage the cavity structure. Thus, the coordination control of the lamination parameters in the final step is very important.

**Figure 6:**
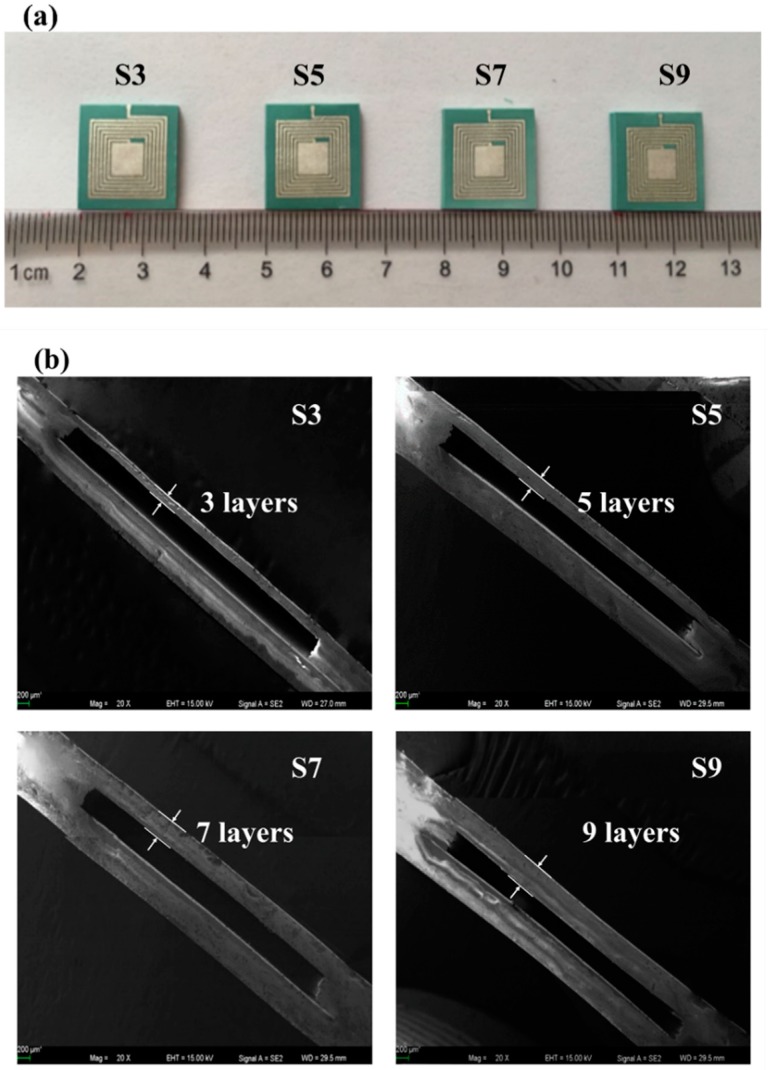
(**a**) Photograph of pressure sensor samples. (**b**) SEM images of the fracture section of the pressure sensors with different sensing membranes thicknesses.

Figure 6a shows the photograph of the wireless pressure sensors. The size of the pressure sensors after the sintering is about 16 × 16 × 1 mm^3^. Figure 6b presents the SEM images of the fracture section of the sensors with different sensing membrane thicknesses. All sensors show a very uniform cavity structure. There is no obvious deformation or crack in the cavity structure. S3, S5, S7, and S9 present sensitive membrane thicknesses of 148 µm, 240 µm, 336 µm, and 432 µm, respectively. Different sensitive membrane thicknesses result in different pressure sensing performances.

**Figure 7:**
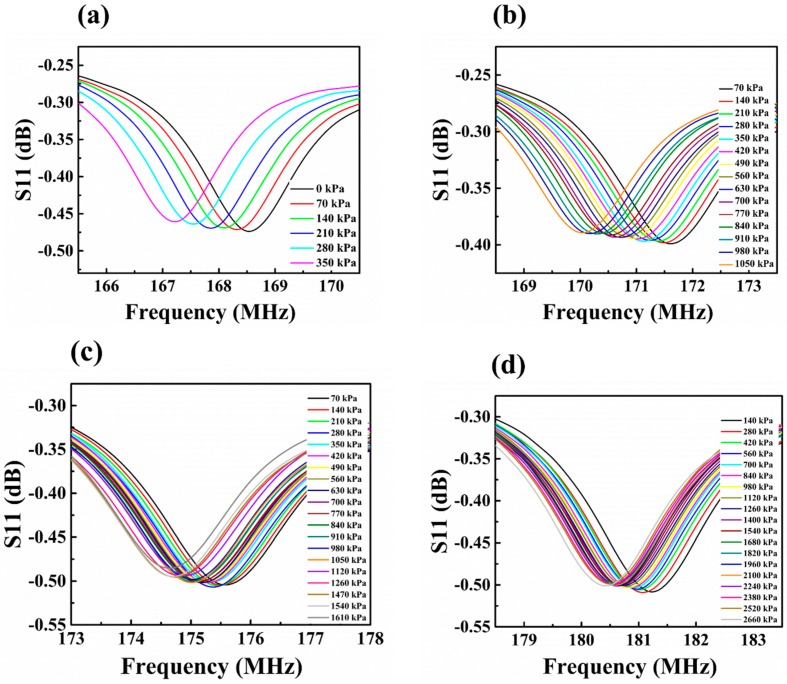
Wireless signal response of the pressure sensors under different pressures: (**a**) S3; (**b**) S5; (**c**) S7; (**d**) S9.

The wireless signal responses of the S3, S5, S7, and S9 pressure sensors under different pressures are shown in Figure 7. It can be seen that, as the sensing membrane thickness increased, both the initial frequency and the pressure detecting range increased. The initial frequencies of S3, S5, S7, and S9 were 168.5 MHz, 171.5 MHz, 175.6 MHz, and 181.1 MHz, respectively. According to Equations (1) and (3), when the thickness of the sensing membrane increased, the capacitance values of the sensor would decrease, and thus the sensor resonant frequency would increase. The pressure detecting limits of S3, S5, S7, and S9 were 350 kPa, 1050 kPa, 1610 kPa, and 2660 kPa, respectively. The increase in pressure detecting limit with sensing membrane thickness is due to the fact that the thicker a sensitive membrane is, the greater a pressure it can endure. It can be also seen that the resonant frequencies of all the sensors decreased with the increases in external pressure. The capacitance variation caused shifts in the resonant frequencies.

**Figure 8:**
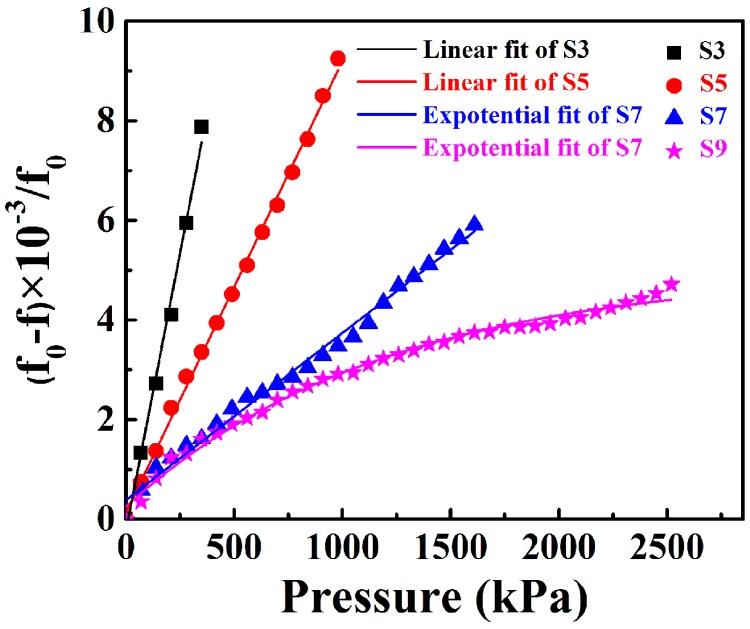
Frequency responses of the four different pressure sensors as a function of pressure.

The response of the four different pressure sensors as a function of pressure is shown in Figure 8. It can be seen that the responses of S3 and S5 increase linearly with the increase in applied pressure. S3 had the highest sensitivity of 3.76 kHz/kPa with a detecting range of 350 kPa. S5 presented a sensitivity of 1.56 kHz/kPa with a detecting range of 1050 kPa, which is higher than the results of previous work [12,14]. The responses of S7 and S9 increase quickly with applied pressure when the applied pressure is low (<400 kPa). However, the increasing trend slows down when the applied pressure is high (>400 kPa). It is noteworthy that S7 and S9 show a high detection range up to 1610 kPa and 2660 kPa, respectively. The tunable pressure detecting range allowed us to fabricate different sensors to meet the different requirements of pressure sensors.

According to membrane deformation theory, when pressure is applied on the sensitive membrane, the varied capacitance can be expressed as [23,24]

(7) $$C_{S}=\frac{C_{0}}{\sqrt{\beta }}\tanh^{-1}\left(\sqrt{\beta }\right)\approx C_{0}\left(1+\frac{\beta }{3}\right)\;$$

(8) $$\beta =\frac{d_{0}}{t_{g}+\frac{t_{m}}{\varepsilon_{r}}}$$

where *C*_0_ is the initial capacitance before deformation, as defined in Equation (3). For the case of the small deflection model of membrane deformation theory, *d*_0_ is directly proportional to *P*, as defined in Equation (6). Thus, Equation (1) can be expressed as

(9) $$f=\frac{1}{2\pi \sqrt{L_{S}C_{S}}}=\frac{1}{2\pi \sqrt{L_{S}C_{0}\left(1+\frac{\beta }{3}\right)}}=f_{0}{\left(1+\gamma P\right)}^{-\frac{1}{2}}$$

(10) $$\gamma =\frac{1}{3}\times \frac{d_{0}}{t_{g}+\frac{t_{m}}{\varepsilon_{r}}}\times \frac{0.00126a^{4}*12\left(1-v^{2}\right)}{E{\left(2t_{m}\right)}^{3}}.$$

Equation (9) can be simplified by the further Taylor expansion:

(11) $$\frac{f}{f_{0}}={\left(1+\gamma P\right)}^{-\frac{1}{2}}=1-\frac{1}{2}\gamma P+\frac{3}{8}\gamma^{2}P^{2}-\cdots \cdots ≅1-\frac{1}{2}\gamma P.$$

Since *γ* is a fixed parameter for a sensitive membrane, it can be concluded that the frequency is linearly related to the pressure. S3 and S5 match the linear relation well. However, the response of S9 shows a typical non-linear characteristic as pressure increases. The non-linear performance of S9 should be caused by the increased thickness of the sensitive membrane. For S9 with a thicker sensitive membrane, the mechanical effect of the membrane itself cannot be ignored. The load *P* would affect the sensitive membrane from two aspects: one is to balance the bending and shearing stress, and the second is to balance the membrane stress. Therefore, the load *P* can be expressed as [24,25]

(12) $$P=\underset{\mathrm{Bending}}{\underset{︸}{\frac{Dd_{o}}{0.0026a^{4}}}}+\underset{\mathrm{Stretching}}{\underset{︸}{{\left(\frac{d_{0}}{0.401a}\right)}^{3}\frac{2Et_{m}}{a}}}.$$

As a result, the deformation *d*_0_ in S9 changes non-linearly with applied pressure *P*.

**Figure 9:**
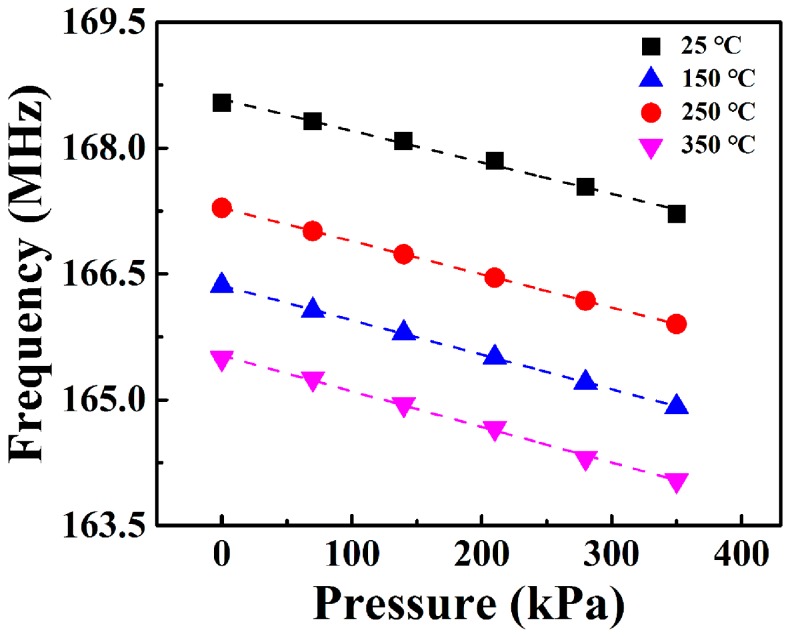
The peak frequency of the sensor versus pressures (0–350 kPa) under different measuring temperatures (25–350 °C).

S3 was selected for investigating the effect of temperature on the pressure measurement. The peak frequency of the sensor versus pressure (0–350 kPa) under different measuring temperatures (25–350 °C) is shown in Figure 9. The response of S3 depends approximately linearly on pressure at elevated temperatures. Under the same loading pressure, the peak frequency of the sensor decreases with the increase in temperature. This should be attributed to the variation of the dielectric constant ($\varepsilon_{r})$ of the LTCC material at elevated temperatures, which resulted in the increase in capacitance. According to Equation (1), the resonant frequency of the sensor decreases as capacitance increases. In addition, the sensitivity of the pressure sensor increases from 3.76 kHz/kPa at 25 °C to 4.25 kHz/kPa at 350 °C. This may be due to the decrease of the Young’s modulus of the LTCC dielectric material as temperature increases, which is in accordance with the reported work [13]. Thus, the temperature compensation should be considered for the real application.

## 5. Conclusions

In this work, passive wireless LC pressure sensors with different sensing membrane thicknesses were investigated. The LTCC material with a low Young’s modulus and the three-step lamination process were used for wireless pressure sensors fabrication. The sensitivities of the pressure sensors decreased as the sensing membrane thickness increased. However, the detection range also increased. The sample with a sensing membrane thickness of 148 μm had the highest sensitivity of 3.76 kHz/kPa, and the sensor with a 432 μm sensing membrane presented a high detection limit of 2660 kPa. This indicated that the pressure measurement range and sensitivity can be controlled by adjusting the sensing membrane thickness of wireless pressure sensors.

## Figures and Tables

**Table 1:** Parameters of the designed LC pressure sensor.

Symbol	Design Value
*d_out_* (mm)	15.5
*d_in_* (mm)	7.75
*w* (mm)	0.5
*s* (mm)	0.25
*n*	6
*a* (mm)	6.52

**Table 2:** The physical parameters of SICCAS-K5F3 and DuPont 951 LTCC materials.

Feature	SICCAS-K5F3	DuPont 951
**Thickness (μm)**	60	50/114/250
**Young’s Modulus (GPa)**	65	120
**Flexural Strength (MPa)**	>200	320
**Dielectric Constant (@10 GHz)**	6.2	7.8
**Dielectric Loss (@10 GHz)**	<0.002	0.005
